# Two anthrax cases with soft tissue infection, severe oedema and sepsis in Danish heroin users

**DOI:** 10.1186/1471-2334-13-408

**Published:** 2013-09-03

**Authors:** Lene Russell, Michael Pedersen, Andreas V Jensen, Lillian Marie Søes, Ann-Brit Eg Hansen

**Affiliations:** 1 Department of Intensive Care 4131, Copenhagen University Hospital, Rigshospitalet, Copenhagen, Denmark; 2 Department of Clinical Microbiology, Copenhagen University Hospital, Rigshospitalet, Copenhagen, Denmark; 3 Department of Infectious Diseases, Hvidovre Hospital, Hvidovre, Denmark; 4 Department of Clinical Microbiology, Hvidovre Hospital, Hvidovre, Denmark; 5 Department of Infectious Diseases, Copenhagen University Hospital, Rigshospitalet, Copenhagen, Denmark

## Abstract

**Background:**

Anthrax had become extremely rare in Europe, but in 2010 an outbreak of anthrax among heroin users in Scotland increased awareness of contaminated heroin as a source of anthrax. We present the first two Danish cases of injectional anthrax and discuss the clinical presentations, which included both typical and more unusual manifestations.

**Case presentations:**

The first patient, a 55-year old man with HIV and hepatitis C virus co-infection, presented with severe pain in the right thigh and lower abdomen after injecting heroin into the right groin. Computed tomography and ultrasonographic examination of the abdomen and right thigh showed oedematous thickened peritoneum, distended oedematous mesentery and subcutaneous oedema of the right thigh. At admission the patient was afebrile but within 24 hours he progressed to severe septic shock and abdominal compartment syndrome. Cultures of blood and intraperitoneal fluid grew *Bacillus anthracis*. The patient was treated with meropenem, clindamycin, ciprofloxacin and metronidazole. Despite maximum supportive care including mechanical ventilation, vasopressor treatment and continuous veno-venous hemodiafiltration the patient died on day four.

The second patient, a 39-year old man with chronic hepatitis C virus infection, presented with fever and a swollen right arm after injecting heroin into his right arm. The arm was swollen from the axilla to the wrist with tense and discoloured skin. He was initially septic with low blood pressure but responded to crystalloids. During the first week, swelling progressed and the patient developed massive generalised oedema with a weight gain of 40 kg. When blood cultures grew *Bacillus anthracis* antibiotic treatment was changed to meropenem, moxifloxacin and metronidazole, and on day 7 hydroxycloroquin was added. The patient responded to treatment and was discharged after 29 days.

**Conclusions:**

These two heroin-associated anthrax cases from Denmark corroborate that heroin contaminated with anthrax spores may be a continuous source of injectional anthrax across Europe. Clinicians and clinical microbiologists need to stay vigilant and suspect anthrax in patients with a history of heroin use who present with soft tissue or generalised infection. Marked swelling of affected soft tissue or unusual intra-abdominal oedema should strengthen clinical suspicion.

## Background

Anthrax, caused by *Bacillus anthracis*, has traditionally been known in three clinical entities depending on the route of entry; cutaneous, gastrointestinal or inhalational anthrax [1]. Twelve years ago Norwegian colleagues described the first case of injectional anthrax associated with heroin use [2]. A recent outbreak of injectional anthrax in Scotland (Dec 2009 - Dec 2010) with more than 80 confirmed and probable cases increased the awareness of this route of infection [3]. This outbreak was also linked to cases in both Germany and England. Very recently, new cases in Germany [4] and other European countries have reinforced the need to suspect anthrax in heroin drug users presenting with signs of infection.

Injectional anthrax has a variety of clinical presentations that differ from the three classical clinical presentations. Generally cases do not present with the typical features of cutaneous anthrax with classical black eschar, but in most cases with marked soft tissue infection with oedema [5] and often with severe pain.

In this article we describe the clinical presentation of the first two Danish cases of anthrax in intravenous heroin users, both of which had some features typical of injectional anthrax, but also demonstrated more rare manifestations.

## Case presentations

### Patient no. 1

#### Day 1

A 55-year old man known with HIV and hepatitis C co-infection and methadone maintenance therapy was admitted with distinct pain and swelling of the right thigh. The patient also complained about intense pain in his right lower abdomen, which on examination was found slightly tense, but not peritoneal. He was afebrile and the C-reactive protein levels in serum were low. Initially he denied having taken any intravenous drugs; later it was revealed that he had injected heroin into the right femoral vein several times during the preceding four days.

An ultrasonographic examination of the right leg performed on suspicion of deep venous thrombosis (DVT) was inconclusive due to scar tissue around the femoral vein. There was subcutaneous oedema but no sign of any abscess. The abdominal ultrasound was similarly inconclusive, although oedema in the bowel walls was seen. A succeeding computed tomography (CT) scan showed a picture initially interpreted as large amounts of ascites or exudate (22–25 Hounsfield Units), but was later re-evaluated as mucous masses, as seen in the rare condition *pseudomyxoma peritonei*. On clinical suspicion of DVT, the patient received low molecular heparin. He was not started on any antibiotics.

During the first night in hospital he complained about abdominal pain. He had pronounced sweating and tachycardia, which was interpreted mainly as opioid abstinence, and he received morphine on several occasions.

#### Day 2

The following morning the patient, after calling the nurse due to vomiting and abdominal pain, collapsed in cardiac arrest with pulseless electrical activity. The cardiac arrest team arrived and spontaneous circulation was re-established after 12 minutes. On suspicion of pulmonary embolus or cerebral insult, a computed tomography (CT) scan of cerebrum and thorax was performed, which were both without abnormality. The patient was started on intravenous antibiotic treatment with cefuroxime 1500 mg q.8.h and metronidazole 500 mg q.8.h on suspicion of aspiration pneumonia.

**Figure 1:**
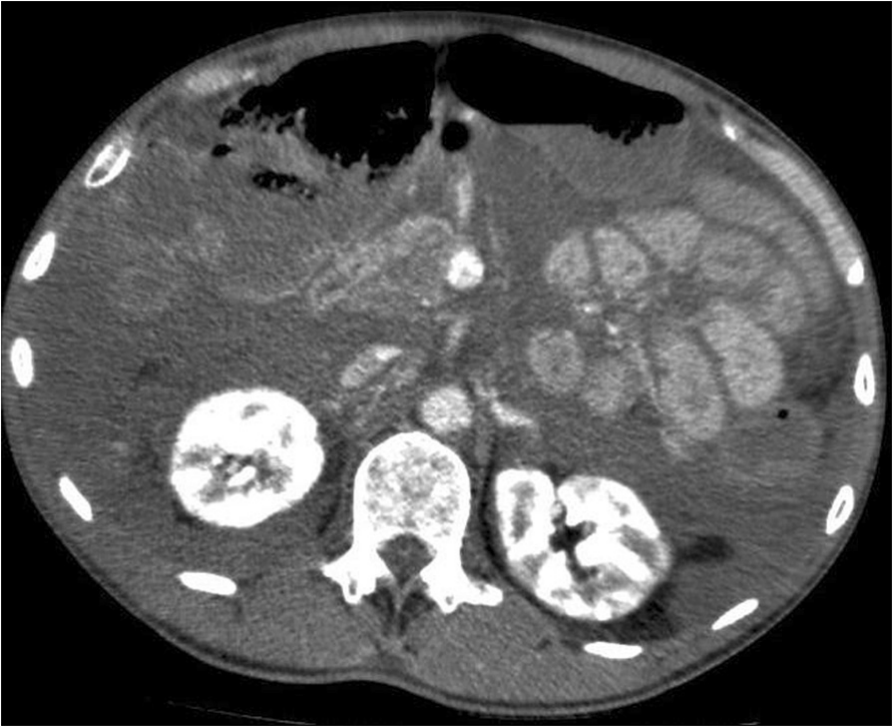
**CT Abdomen of patient 1, here at kidney–level, showing the dense mucous masses initially interpreted as pseudomyxoma peritonei filling the abdominal cavity, making the individual organs difficult to see.** On this second CT-scan from day 2, the masses are increasingly dense, now measuring around 50 HU. There are no obvious signs of bowel ischemia.

**Figure 2:**
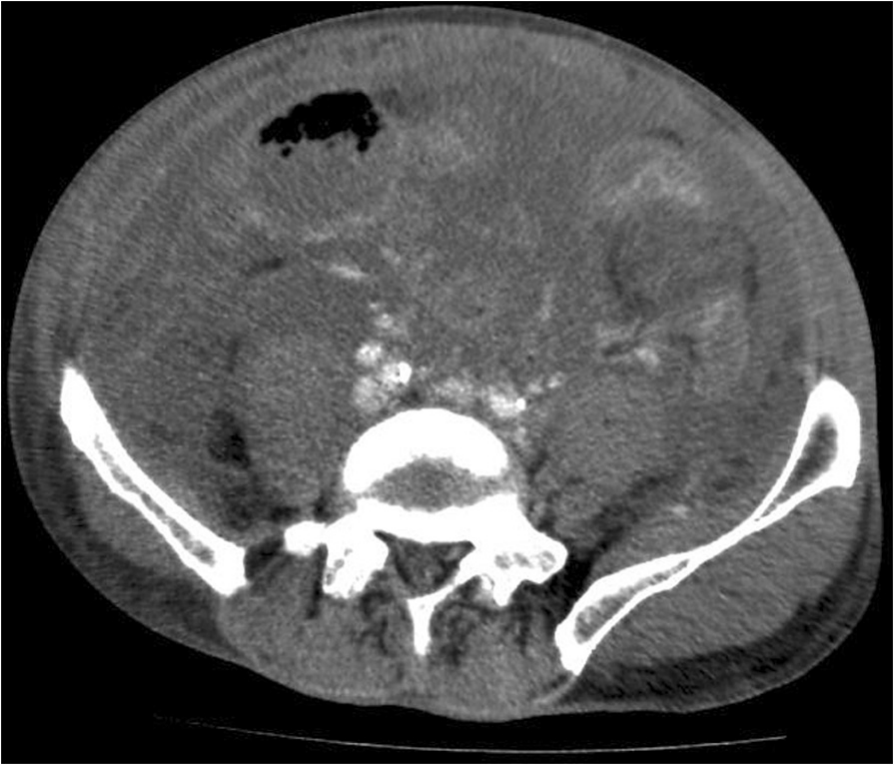
CT abdomen (patient 1), pelvic level, where the severely oedematous peritoneum and mesentery are filling the abdominal cavity.

**Figure 3:**
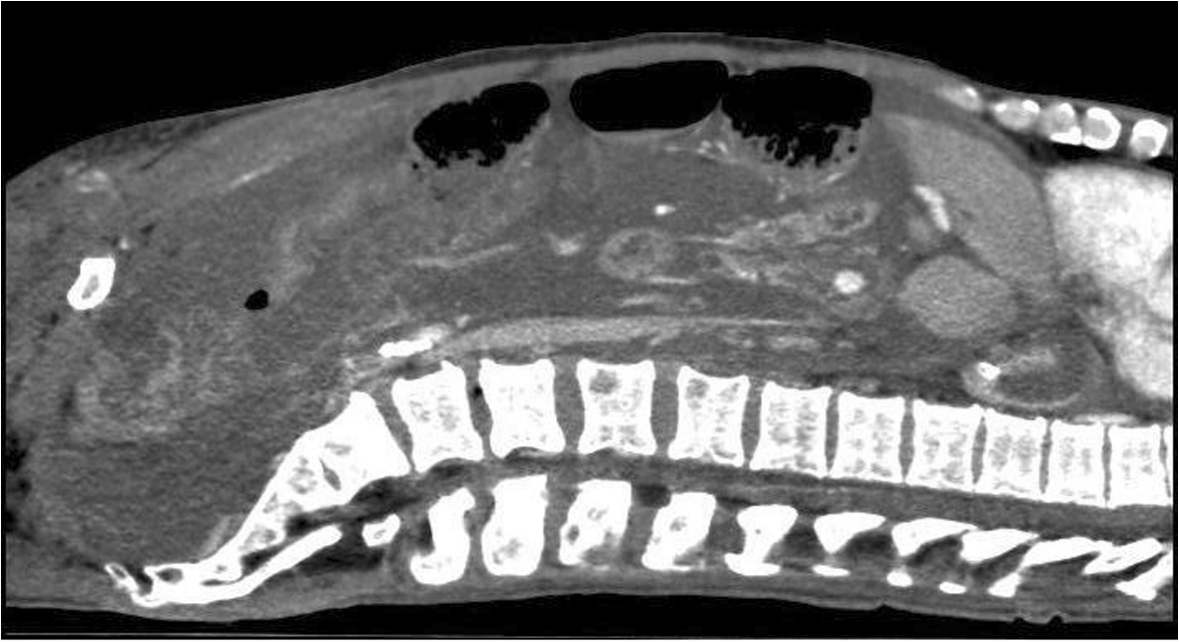
CT abdomen (patient 1), sagittal view with contrast, again illustrating what is seen as mucous masses filling the abdominal cavity, but which by ultrasonographic examination was revealed as massively thickened peritoneum.

He was transferred to the Intensive Care Unit where he expressed fulminant septic shock with multi-organ failure needing immediate fluid resuscitation, assisted ventilation and high doses of noradrenaline. He was severely hypothermic with a core temperature of 35 degrees Celsius, blood pressure 84/52 mmHg and mean arterial pressure of 61 mmHg on noradrenalin infusion of 0.2 microgram per kilogram per minute. His arterial blood gas analysis showed severe metabolic acidosis, pH 6.99, standard base excess −16, and plasma lactate concentration of 10.1 millimoles per litre. He required a FiO2 of 1.0 to keep PaO2 over 8 kPa. There were no secretions or mucus in the trachea or bronchus and no pleural effusions, but severe oedema of the lungs on chest x-ray. The cardiac status was assessed to be normal using trans-oesophageal echocardiography. The disseminated intravascular coagulation (DIC)-score was 6 [6]. He had a rigid peritoneal abdomen wall and a swollen, upper right leg. The abdominal rigidity and intra-abdominal pressure increased in the following hours. The swelling of the abdominal wall was moderate, but the patient had severe abdominal compartment syndrome with intra-abdominal pressure of 25 mmHg. A new computed tomography (CT) scan of the abdomen was performed which showed increasing masses of suspected mucus with no signs of ileus or perforation (Figures 1, 2 and 3). An ultrasound-guided needle aspiration was performed; the ultrasound revealed that the fluid seen on the CT scan represented thickened peritoneum and an abnormally distended oedematous mesentery. There were only insignificant amounts of intraperitoneal fluid. Nevertheless, two samples of slightly milky fluid were extracted and sent for microbiological analysis. The abdominal surgeon concluded that surgical intervention was not possible.

The antibiotic regime was changed to meropenem 1g q.8.h, ciprofloxacin 400 mg q.2.h and metronidazole 500 mg q.8.h. As the patient was anuric, acidotic, had received large amounts of intravenous crystalloid infusions and seemed vastly over hydrated, continuous veno-venous hemodiafiltration (CVVHDF) was started.

During the following 12 hours the condition of the patient slightly improved, with FiO2 decreasing to 0.8 and the intra-abdominal pressure falling to 14 mmHg. He regained consciousness and responded to verbal commands (Glasgow Coma Scale 12).

#### Day 3

After the initial recovery seen after the change of antibiotic treatment and commencement of CVVHDF, the patient’s status again deteriorated as he developed hepatic failure, rhabdomyolysis, increasing coagulopathy and acidosis. He was started on treatment with intravenous steroids in a last attempt to reverse the rapid progression of shock.

When blood cultures revealed gram-positive rods the meropenem and ciprofloxacin dose was increased to 2 gram q.8.h. and 400 mg q.8.h. respectively, and clindamycin 600 mg q.8.h. was added on suspicion of *Bacillus anthracis* infection. The abdominal and orthopaedic surgeons were again consulted, but surgical intervention was declined due to the patient’s unstable condition and the absence of abscesses.

#### Day 4

The patient died while on maximum treatment with ventilator support, vasopressor and CVVHDF.

### Patient no. 2

#### Day 1

A 39-year old man with chronic hepatitis C virus infection and intravenous usage of heroin presented at the Emergency department with a swollen right arm and fever. At admission the blood pressure was 77/44 mmHg, pulse 110 beats/min, temperature 39.3°C and arterial oxygen saturation 96 %. He was somnolent. The right arm was swollen from the axillary region to the wrist and the skin was tense and discoloured.

The initial suspicion was, as in the first case, soft tissue infection or deep venous thrombosis. He was treated with cefuroxime 1500 q.8.h. and given dalterapin. Fluid treatment with isotonic saline normalised the blood pressure. The orthopaedic surgeon was consulted on the suspicion of compartment syndrome, on which no basis was found.

#### Day 2

The following day the patient was able to communicate that he had injected brown heroin in his right arm. He was not able to specify when he had injected the substance.

An ultrasonographic examination of the arm showed no sign of thrombosis, but as in the first case, diffuse soft tissue swelling was seen. The patient’s blood culture showed growth of gram-positive rods and on suspicion of infection with *Bacillus anthracis* the patient was started on intravenous treatment with meropenem 2 g q.8.h., metronidazole 500 mg q.8.h. and moxifloxacine 400 mg q.d.. Moxifloxacine was chosen to ensure as broad-spectrum coverage as possible until confirmed microbiological identification. He was admitted to the Infectious Diseases department for further treatment.

#### Day 3 – 10

The swelling of the right arm progressed to involve most of the body. His blood pressure was fluctuating with episodes of systolic blood pressure dropping to 60 mmHg, however kidney function was preserved and fluid treatment with crystalloid and albumin was sufficient to stabilize the blood pressure. At day 7, treatment with hydroxychloroquine 500 mg q.d. was initiated, followed by 250 mg q.d. every third day for a total of 7 days in an attempt to inhibit the effect of the *B. anthracis* toxins.

As the alanine aminotransferase levels and International normalised ratio (INR) were increasing, the antibiotic treatment was changed to clindamycin 600 mg q.8.h. and ciprofloxacin 400 mg q.12.h.. On day 9, extended susceptibility testing of *B. anthracis* showed sensitivity to penicillin, so antibiotic treatment was changed to benzyl penicillin 1200 mg q.4.h. and ciprofloxacin 400 mg q.12.h.

#### Day 11 – 29

The patient’s condition improved rapidly and the blood pressure stabilised. He received intravenous antibiotic treatment for 17 days, followed by 4 days treatment with amoxicillin-clavulanate pills. The patient’s main problem during this period was the substantial oedema. At day 15 he weighed 108 kg, which was 40 kg more than his habitual weight. The following days he was treated with compression bandages. The patient continued to improve and when discharged on day 29 his weight was normalised.

Standard infection control precautions were applied in both cases. Patients were placed in singe rooms and health care staff used gloves and gowns. In case 1 standard surgical masks were used.

### Laboratory analysis

#### Patient no. 1

**Figure 4:**
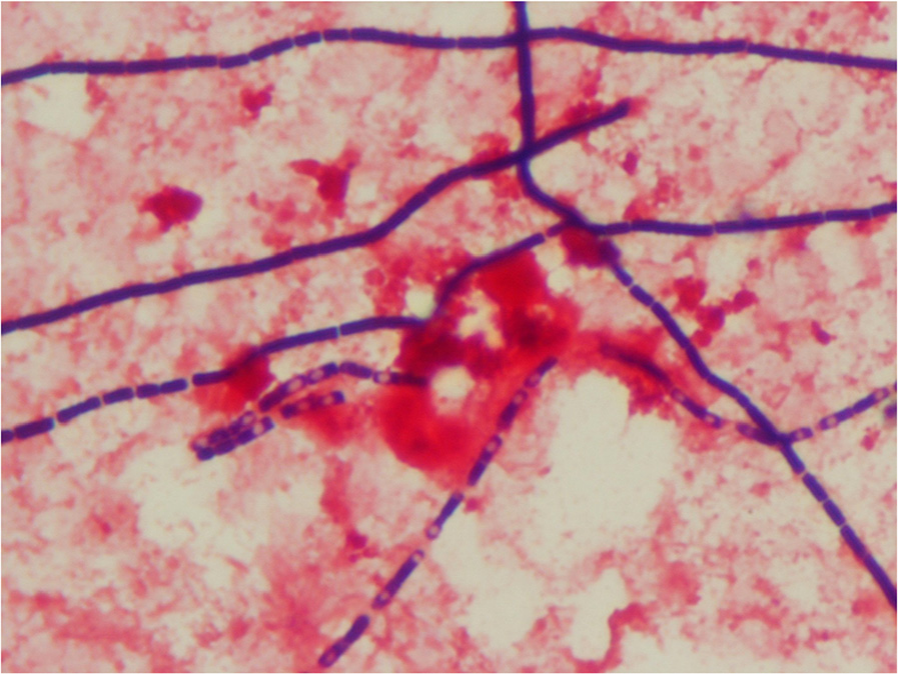
**Gram stain of *B. anthracis*, showing large gram-positive rods with sub terminal ellipsoidal spores in blood culture from patient 1.**

*Bacillus anthracis* was found in both aerobic blood culture (Becton Dickinson, Heidelberg; Germany) and in culture from ascites fluids on a 5 per cent horse blood agar after 22 to 24 hours of incubation at 35°C. Phase-contrast microscopy showed large non-motile bacilli and Gram stain revealed non-branching gram-positive bacilli growing in chains (Figure 4).

On subcultures the bacilli were without haemolysis and were lipase negative on egg yolk agar. Matrix-assisted laser desorption/ionization time-of-flight mass spectrometry (Bruker Daltonics, Bremen, Germany) was performed. The patient history led to analysis with the Bruker database for security-relevant organisms. The organism was correctly identified as *Bacillus anthracis*. From the blood the score value for best match organism was 2.568 un-extracted and 2.335 with formic acid extraction. From ascites the un-extracted score was 2.324.

Susceptibility testing was performed. The minimal inhibitory concentrations (Etest, AB bioMérieux, Solna, Sweden) were azithromycin = 2.0 μg/mL, ciprofloxacin = 0.047 μg/mL, clindamycin = 0.5 μg/mL, meropenem = 0.064 μg/mL and penicillin = 0.094 μg/mL.

A relative of the patient confirmed that the heroin was bought locally in Copenhagen and handed in samples of the remaining heroin. The samples of heroin were analysed at the National Centre for Biological Defence, Statens Serum Institute, but *B. anthracis* spores were not detected (5).

#### Patient no. 2

Large non-motile gram-positive bacilli arranged end to end in chains were found in the aerobic blood cultures (BacT/ALERT, bioMérieux, France). Both culture and PCR for *B. anthracis* from the wound were negative.

The isolate was sent the National Reference Laboratory, Statens Serum Institute. Susceptibility testing was performed using disk diffusion test. The isolate was susceptible to meropenem, penicillin, clindamycin, vancomycin, tetracycline, ciprofloxacin and moxifloxacin, but resistant to rifampicin. The minimal inhibitory concentration for penicillin was 0.064 μg/mL.

Isolates from both cases were sent to the Robert Koch Institute in Berlin. A TaqMan based multiplex qPCR for *B. anthracis* was performed. All three chromosomal markers, *pag* (pXO1), *capB* (pXO2) and *rpoB* were detected by the PCR. In addition the chromosomal marker *dhp61* was found after DNA isolation. Comparative molecular typing with a high resolution 31 marker multilocus variable-number tandem repeat analysis (MLVA) and a broad single nucleotide polymorphism (SNP) analysis revealed that the isolates were identical to the *B. anthracis* isolates from earlier outbreaks among injection drug users [7]. For patient no. 2, an ELISA for *B. anthracis* antibodies on a serum sample was negative although the titer was higher than the control.

## Discussion

In this article we describe the first two cases of injectional anthrax in Denmark. They both presented with marked pain and oedema of the affected tissue, which is consistent with earlier descriptions in literature [5,8]. The molecular typing of these two bacterial isolates has recently been described by Grunow *et al.*[7].

The first patient presented with pain in the thigh and abdomen and with rapid progression to systemic involvement. He died on day 4. The second patient, who presented with fever and a swollen arm, developed a generalised oedema of approximately 40 kg and although he initially was septic with low systolic blood pressure and affected mental status, he responded well to the given treatment and was discharged after 29 days of hospitalisation. These two cases illustrate how essential it is to start antibiotic therapy promptly. Both patients presented with similar symptoms. However, the first patient did not receive relevant antibiotic treatment until 24 hours after the symptoms appeared and by that stage, the infection had progressed into intractable septic shock, whereas in the second patient antibiotic treatment was initiated early, and his symptoms were halted and he was managed entirely outside the intensive care environment.

The first patient had unusual gastrointestinal findings, very similar to one previous published case [9]. Initially, after the first computed tomography (CT) scan, the radiologists described the findings as large amounts of ascites or mucus masses. Abdominal ultrasound examination later revealed that the findings represented thickened peritoneum and an oedematous and distended mesentery. Gastrointestinal symptoms have been described to predominate in some cases of injectional anthrax and are probably reflecting disseminated disease. In Scotland, abdominal pain was noted in 46% of confirmed and 17% of probable cases. Despite the abdominal symptoms, the patient was afebrile and hemodynamically stable at admission, which is in line with another reported case by Powell *et al.*[10], but within less than 24 hours he collapsed in cardiac arrest. Very rapid deterioration has been noted in several previously described cases, and it is well known that patients with systemic disease have a very poor prognosis [1].

The main virulence factors of *Bacillus anthracis* are the two toxins: Lethal Toxin (LeTx) and Edema toxin (ETx). They are both binary proteins coupled with a protective antigen and are implicated in the pathogenesis of shock and rapid cell-death [11]. There are still questions left to answer regarding the underlying mechanism of the toxins [12,13]. Lawrence *et al.*[14] showed in a rabbit model that the lethal toxin (LeTx) might have direct cardiotoxic effects and it is possible that toxins caused the early cardiac arrest in the first patient. Anti-toxin therapies are under investigation and are considered experimental [15]. Chloroquine is known to interfere with the lysosomale processing of LeTx [16] and chloroquine has been shown to have a positive effect on the survival of mammalian cells *in vitro* as well as in experimental studies of mice injected with LeTx [17]. The second patient was treated with chloroquine with a possible positive effect, but the treatment is experimental and further studies are needed.

It is generally agreed that patients with septic shock from *Bacillus anthracis* should be treated with aggressive hemodynamic support [1] and the patient in the first case was indeed treated with large amounts of intravenous crystalloid solution as well as noradrenalin due to circulatory failure. However, experimental animal studies indicate that aggressive fluid therapy can be less beneficial in anthrax [18] and in rats exposed to LeTx, noradrenalin increases the blood pressure but does not enhance survival [19]. It is possible that a more restrained fluid approach would have been useful. It was noteworthy how rapid the abdominal compartment syndrome progressed during the first hours after ICU admission and there was actually an initial positive response with a decrease of intra-abdominal pressure after starting the continuous veno-venous hemodiafiltration (CVVHDF ). The patient tolerated fluid removal through hemofiltration surprisingly well and his condition actually briefly improved. In the latter stages of anthrax treated with relevant antibiotics, disease progression is mediated by the effects of the toxins. Due to the size of anthrax toxins, removal through dialysis is not possible.

Epidemiological evidence implicating heroin as the vehicle for spore transmission during the Scottish outbreak was very strong. Our case together with the recent cases in Germany, Great Britain and France suggest that contaminated heroin may be a continuous source of anthrax in Western Europe leading to both outbreaks and sporadic cases. It is assumed that the contamination with anthrax spores occurs during transportation from Afghanistan through Turkey to Western Europe and it has been speculated that the contamination might be through contact with goatskins or bonemeal [20]. No *Bacillus anthracis* spores were detected in the samples of heroin that were handed in by the patient’s relative. This is similar to the Scottish outbreak, where investigators also failed to detect anthrax in collected heroin samples [5], indicating that the concentration of spores in the heroin was very low.

## Conclusion

In conclusion, clinicians and clinical microbiologists need to stay vigilant and suspect anthrax in heroin drug users presenting with soft tissue infection as well as systemic infection and sepsis. As our second case illustrates injectional anthrax can be successfully treated with antibiotics, especially if treatment is commenced before development of severe systemic involvement. Once *Bacillus anthracis* infection has developed into septic shock it should be treated aggressively with hemodynamic and ventilator support.

## Consent

Written informed consent for the publication of this case report was obtained was from the patient in the second case and from the closest relative (brother) of the patient in the first case. Copies of both documents are available for review.

## Competing interests

The authors declare that they have no competing interests.

## Authors’ contributions

LR was involved in the treatment of patient no. 1, carried out the literature review, the acquisition and interpretation of data, prepared the figures and drafted and critically revised the manuscript. MP was responsible for analysis and interpretation of laboratory data, drafting and revising the manuscript as well as helping with the figures. AVJ was involved in the treatment of patient no. 2 and drafting the parts of the manuscript involving this case. LMS contributed to microbiological analysis of the isolate and handling of patient specimen in case 2 and helped with revision of the manuscript. ABEH was involved in the treatment of the patient no. 1, carried out the literature review, the acquisition and interpretation of data, coordinated, drafted and critically revised the manuscript. All authors read and approved the final manuscript.

## Pre-publication history

The pre-publication history for this paper can be accessed here:

http://www.biomedcentral.com/1471-2334/13/408/prepub
